# Understanding the Complex Relationship Between Gastrointestinal Symptoms and Psychosocial Factors in Schizophrenia

**DOI:** 10.1192/j.eurpsy.2024.1554

**Published:** 2024-08-27

**Authors:** W. Krzyściak, M. Szwajca, N. Śmierciak, A. Turek, M. Pilecki

**Affiliations:** ^1^Department of Medical Diagnostics; ^2^Department of Child and Adolescent Psychiatry, Jagiellonian University, Medical College, Krakow, Poland

## Abstract

**Introduction:**

Insensitivity to pain in schizophrenia is a complex phenomenon. Understanding schizophrenia’s heterogeneity is crucial for personalized treatments.

**Objectives:**

Individuals diagnosed with schizophrenia often experience gastrointestinal issues and exhibit elevated levels of depression and anxiety. There is an urgent need to understand how these factors interact and how childhood traumas, a significant risk factor for schizophrenia, can affect gastrointestinal symptoms in these individuals.

**Methods:**

The study involved 51 individuals diagnosed with schizophrenia. The hierarchical cluster analysis on the principal components (HCPC) was performed to identify groups of similar observations for test scores and the overall results for 14 tests. Hierarchical clustering was performed using Ward’s minimum variance method. Differences in the results of individual tests between clusters were estimated using the *V* test.

**Results:**

The schizophrenia group was categorized into three clusters. The patients belonging to the first cluster are characterized by high GAF test scores and low scores on tests for gastrointestinal symptoms, ITQ, CTQ, GHQ-28, STAI, CALGARY, BDI II, SAMPS, SANS, and PANNS. In contrast, patients in the second cluster had scores significantly above the group average on the tests SANS, PANNS, and SAPS and low scores on the tests DBZ RZ, CTQ, STAI, BDI II, ITQ, and GAF. Finally, patients in the third cluster had high scores on the tests BDI II, ITQ, STAI, CTQ, GHQ 28, DBZ RZ, gastrointestinal symptoms, TEC PL, CALGARY, and CISS. High CTQ scores may contribute to increased GSSR scores due to childhood trauma’s potential to trigger chronic stress, affect the nervous system, and induce psychosomatic symptoms, including gastrointestinal problems. Elevated BDI II and STAI scores can also impact GSSR results by disrupting the connection between emotions and the gastrointestinal system.

**Conclusions:**

This research underscores the intricate interplay of various psychosocial and physiological factors that influence the perception of pain related to gastrointestinal symptoms in individuals with schizophrenia.

**Disclosure of Interest:**

None Declared

